# Spatial Immune Remodeling Across the Gallbladder Carcinogenesis Spectrum: A Multicenter Digital Pathology Study

**DOI:** 10.14740/wjon2771

**Published:** 2026-06-25

**Authors:** Tamta Kveliashvili, George Didava, George Burkadze, Shota Kepuladze

**Affiliations:** a Department of Pathology, Tbilisi State Medical University, Tbilisi, Georgia

**Keywords:** Gallbladder carcinoma, Tumor immune microenvironment, CD8, FOXP3, CD163, Tumor-associated macrophages, Immune exclusion, Digital pathology, Biliary intraepithelial neoplasia

## Abstract

**Background:**

Gallbladder carcinoma (GBC) is the most aggressive malignancy of the biliary tract and is commonly associated with late diagnosis and early metastatic dissemination. Although the morphological sequence of gallbladder carcinogenesis from chronic inflammation to epithelial dysplasia and invasive carcinoma is well recognized, the role of the tumor immune landscape during this process remains insufficiently characterized. This study aimed to quantitatively evaluate immune microenvironment alterations across the spectrum of gallbladder lesions using immunohistochemistry and digital pathology analysis.

**Methods:**

A retrospective multicenter study was conducted using formalin-fixed paraffin-embedded gallbladder specimens collected between 2015 and 2026 from several collaborating hospitals and analyzed at the Department of Molecular Pathology, Tbilisi State Medical University. The study cohort included 90 cases representing chronic cholecystitis, intestinal metaplasia, biliary intraepithelial neoplasia (BilIN-1, BilIN-2, BilIN-3), and invasive gallbladder adenocarcinoma. Immunohistochemical analysis was performed for CD8 (cytotoxic T lymphocytes), FOXP3 (regulatory T cells), and CD163 (tumor-associated macrophages). Whole-slide digital image analysis using QuPath and ImageJ was applied to quantify immune cell densities and evaluate spatial distribution between tumor center and invasive tumor front compartments. Composite immune indices were calculated to integrate cytotoxic and immunosuppressive immune populations. The immune suppression index was defined as (FOXP3 + CD163)/CD8.

**Results:**

Quantitative analysis demonstrated progressive remodeling of the immune landscape across the spectrum of gallbladder lesions. CD8-positive cytotoxic lymphocytes were present in all lesion categories but showed heterogeneous density in invasive carcinoma. In contrast, FOXP3-positive regulatory T cells and CD163-positive macrophages demonstrated increased infiltration in dysplastic lesions and invasive tumors. Spatial analysis revealed preferential localization of CD8-positive lymphocytes at the invasive tumor margin with reduced infiltration of the tumor center, consistent with an immune exclusion pattern. Tumors associated with liver metastases exhibited lower cytotoxic lymphocyte density and increased infiltration by regulatory T cells and macrophages. Composite immune suppression indices were correspondingly elevated in metastatic tumors.

**Conclusions:**

Gallbladder carcinogenesis is associated with significant remodeling of the tumor immune microenvironment characterized by enrichment of immunosuppressive immune populations and spatial restriction of cytotoxic lymphocyte infiltration. Digital pathology-based quantification of immune architecture provides reproducible assessment of tumor–immune interactions and may contribute to the identification of immune-related biomarkers of tumor aggressiveness in GBC.

## Introduction

Gallbladder carcinoma (GBC) represents the most common malignancy of the biliary tract and remains among the most aggressive gastrointestinal cancers [1]. Despite advances in surgical techniques and diagnostic imaging, prognosis remains poor, largely because the disease is frequently detected at advanced stages [2]. Gallbladder carcinogenesis is generally considered a multistep process that begins with chronic inflammation and progresses through epithelial alterations including metaplasia and biliary intraepithelial neoplasia (BilIN) before ultimately evolving into invasive adenocarcinoma [3].

Although morphological progression of gallbladder lesions has been extensively characterized, the biological mechanisms that facilitate tumor progression and metastatic dissemination remain incompletely understood. Increasing evidence indicates that tumor development is not determined solely by intrinsic genetic alterations of epithelial cells but also by interactions with the surrounding tumor microenvironment [4, 5].

Among the components of the immune landscape, immune cells play a central role in regulating tumor growth, invasion, and metastasis. Cytotoxic CD8-positive T lymphocytes represent the primary mediators of antitumor immunity and their presence in hepatobiliary tumors has been associated with improved survival and better immune control of tumor progression [6, 7]. In multiple malignancies, increased infiltration of CD8-positive lymphocytes correlates with improved patient outcomes and enhanced response to immunotherapy. Conversely, tumors with reduced cytotoxic immune infiltration often demonstrate immune evasion mechanisms and aggressive biological behavior [8].

Tumor immune escape is frequently associated with expansion of immunosuppressive immune cell populations. Regulatory T cells characterized by FOXP3 expression suppress cytotoxic immune responses and contribute to immune tolerance within the immune landscape. In addition, tumor-associated macrophages with an M2-like phenotype, often identified by CD163 expression, promote tumor progression by supporting angiogenesis, extracellular matrix remodeling, and suppression of cytotoxic T-cell activity [9].

Beyond immune cell composition, the spatial organization of immune infiltrates has emerged as a critical determinant of tumor–immune interactions. In several solid tumors, cytotoxic lymphocytes accumulate at the invasive tumor margin but fail to effectively infiltrate the tumor core. This phenomenon, known as immune exclusion, reflects impaired penetration of antitumor immune cells into tumor tissue and has been associated with tumor immune escape [10].

Despite increasing recognition of the role of tumor–immune interactions in cancer progression, the immune landscape of gallbladder lesions remains insufficiently characterized. In the present study, composite immune indices integrating cytotoxic and immunosuppressive immune populations were evaluated in relation to clinicopathological features and metastatic behavior. Digital whole-slide image analysis was additionally applied to quantify immune cell densities and spatial distribution across tumor compartments. The present multicenter study investigates immune microenvironment remodeling across the spectrum of gallbladder lesions using immunohistochemical markers of cytotoxic lymphocytes (CD8), regulatory T cells (FOXP3), and tumor-associated macrophages (CD163). Using digital whole-slide image analysis, immune cell densities and spatial distribution within tumor compartments were quantified. Composite immune indices integrating cytotoxic and immunosuppressive immune populations were evaluated to explore potential associations with clinicopathological features and metastatic behavior.

Unlike most previous studies focusing solely on invasive carcinoma, the present investigation analyzes immune landscape changes across the full spectrum of gallbladder carcinogenesis, including inflammatory lesions, precursor epithelial alterations, and invasive tumors.

This design allows evaluation of immune remodeling during early stages of tumor development.

In addition, spatial digital pathology analysis was used to quantify immune cell distribution between tumor center and invasive tumor front compartments. Unlike most previously published studies focusing exclusively on invasive GBC, the present study evaluates immune remodeling across the complete pathological spectrum of gallbladder carcinogenesis using spatial digital pathology analysis. This approach enables characterization of early immune alterations occurring during progression from inflammatory and precursor lesions to invasive malignancy.

## Materials and Methods

### Study design and case selection

This retrospective multicenter study was conducted at the Department of Molecular Pathology, Tbilisi State Medical University (TSMU), Tbilisi, Georgia. The department receives surgical pathology specimens from multiple collaborating hospitals located in Tbilisi, Batumi, and Kutaisi, allowing the construction of a multicenter pathology cohort.

Formalin-fixed paraffin-embedded (FFPE) gallbladder specimens archived between 2015 and 2026 were retrieved from the institutional pathology archive. Cases were reviewed and classified into diagnostic categories representing the spectrum of gallbladder pathology, including chronic cholecystitis, intestinal metaplasia, BilIN (BilIN-1, BilIN-2, BilIN-3), and invasive gallbladder adenocarcinoma.

Histological diagnoses were confirmed by review of hematoxylin and eosin (H&E)-stained slides. Cases with insufficient epithelial tissue, severe fixation artifacts, or extensive cautery damage were excluded. Clinical and follow-up data were obtained from pathology reports and available medical documentation.

The final study cohort included 90 cases, representing inflammatory lesions, precursor epithelial alterations, and invasive carcinoma.

The study was conducted in accordance with the Declaration of Helsinki and approved by the Ethics Committee of TSMU (Approval No. TSMU-MP-2022-017). The study used archived pathological material and anonymized clinical data; therefore, individual patient consent was waived.

### Clinicopathological data collection

Clinical and pathological parameters were extracted from the pathology database and clinical records. Recorded variables included patient age at diagnosis, sex, year of surgery, presence of cholelithiasis, specimen type, and final histopathological diagnosis.

For carcinoma cases, additional clinicopathological parameters were documented, including tumor grade, pT stage, pN stage, lymphovascular invasion, perineural invasion, surgical margin status, tumor budding, tumor area, tumor cellularity, and necrosis. Follow-up variables included recurrence status, time to recurrence, liver metastasis, follow-up duration, and vital status when available. These clinicopathological variables were used for subgroup comparisons and correlation analysis with immune microenvironment parameters, including immune cell densities, spatial immune distribution, and composite immune suppression indices.

### Immunohistochemistry (IHC)

Immunohistochemical analysis was performed on 4-µm FFPE tissue sections using a Leica automated staining platform with polymer-based detection chemistry according to manufacturer protocols.

The antibody panel included markers representing epithelial phenotype, epithelial–mesenchymal transition, proliferation, and immune microenvironment (Table 1). Appropriate positive and negative controls were included in each staining run to ensure staining quality and reproducibility.

**Table 1:** Immunohistochemical Antibodies Used in the Study

Marker	Clone	Dilution	Localization
E-cadherin	NCH-38	1:100	Membranous
β-catenin	E247	1:100	Membranous/nuclear
Vimentin	V9	1:200	Cytoplasmic
CK17	E3	1:100	Cytoplasmic
CK19	A53-B/A2	1:150	Cytoplasmic
CDX2	EPR2764Y	1:100	Nuclear
Ki-67	MIB-1	1:200	Nuclear
p53	DO-7	1:100	Nuclear
FOXP3	236A/E7	1:100	Nuclear
CD8	C8/144B	1:100	Membranous
CD163	10D6	1:100	Cytoplasmic
HER2	4B5	Ready-to-use	Membranous

List of primary antibodies applied for immunohistochemical analysis, including marker name, antibody clone, working dilution, and expected subcellular localization of staining. The panel included epithelial markers (E-cadherin, β-catenin, CK17, CK19), mesenchymal marker (vimentin), intestinal differentiation marker (CDX2), proliferation marker (Ki-67), tumor suppressor protein (p53), immune markers (FOXP3, CD8, CD163), and HER2 receptor status.

### Whole-slide imaging and digital pathology analysis

**Figure 1:**
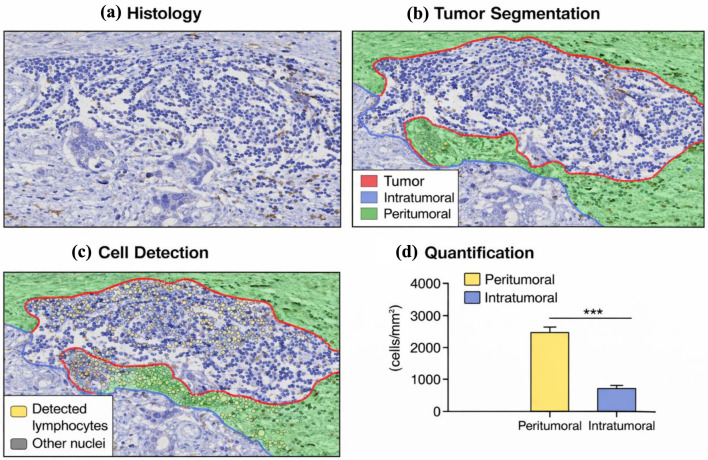
Spatial digital analysis of immune cell infiltration in gallbladder adenocarcinoma. (a) Representative high-power field (× 40) showing tumor glands with surrounding lymphoid infiltrate. (b) Digital segmentation distinguishing tumor and stromal compartments. (c) Automated lymphocyte detection using digital pathology analysis. (d) Quantification of immune cell density demonstrating higher lymphocyte accumulation in the peritumoral region compared with the intratumoral compartment.

Slides were digitized at × 40 equivalent magnification (0.25 µm/pixel resolution). Digital image analysis was performed using QuPath software and ImageJ software (Fig. 1).

Regions of interest (ROIs) were defined on digital slides based on corresponding H&E morphology. The invasive tumor front was defined as the peripheral tumor region directly interacting with adjacent stromal tissue, whereas the tumor center represented deeply located intratumoral areas without direct stromal interface. For each case, representative regions covering approximately 1–2 mm^2^ of tissue were analyzed. In carcinoma cases, two tumor compartments were evaluated.

Areas containing necrosis, hemorrhage, folds, or staining artifacts were excluded from analysis. Immune cell densities were quantified automatically and verified by manual review. Digital cell detection parameters in QuPath were standardized across all cases using hematoxylin optical density thresholding and automated positive cell detection algorithms. Manual verification was performed for representative regions to ensure segmentation accuracy.

Immune cell infiltration was expressed as cells per square millimeter (cells/mm^2^). Spatial immune parameters included measurements of immune cell densities separately within tumor center and invasive front compartments (Table 2).

**Table 2:** Estimated Lymphocyte Counts and Immune Cell Density Derived From Digital Spatial Analysis of Representative Tumor Regions

Region	Lymphocytes (n)	Area (mm^2^)	Density (cells/mm^2^)
Peritumoral compartment	∼320	∼0.08	∼4,000
Intratumoral compartment	∼80	∼0.08	∼1,000

### Quantification of immunohistochemical markers

Immunohistochemical marker expression was evaluated using quantitative and semi-quantitative approaches.

Proliferative activity was assessed using Ki-67 labeling index, expressed as percentage of positive tumor nuclei. Expression of epithelial and epithelial–mesenchymal transition (EMT) markers (E-cadherin, β-catenin, vimentin) was assessed using H-score methodology ranging from 0 to 300.

Cytokeratin expression (CK17, CK19) and intestinal differentiation marker CDX2 were recorded as the percentage of positive tumor cells.

Immune cell densities were quantified digitally for CD8-positive cytotoxic T lymphocytes, FOXP3-positive regulatory T cells, and CD163-positive macrophages.

To integrate immune microenvironment parameters, composite indices were calculated.

The Immune Index was defined as the relative balance between cytotoxic and immunosuppressive immune cell populations. The immune suppression index was designed to reflect the relative predominance of immunosuppressive immune populations (FOXP3-positive regulatory T cells and CD163-positive macrophages) over cytotoxic CD8-positive lymphocytes within the tumor microenvironment.

To integrate immune cell populations into a single quantitative parameter, an immune suppression index was calculated using the formula: Immune suppression index = (FOXP3 density + CD163 density)/CD8 density.

In addition, spatial immune measurements were used to assess differences between tumor center and invasive front compartments, representing patterns of immune infiltration within the tumor microenvironment.

Statistical analysis was performed using SPSS software (version 21) and verified using statistical packages in Python. Continuous variables were summarized as median with interquartile range (IQR) due to non-normal distribution of immune cell densities. Categorical variables were expressed as frequencies and percentages. Comparisons between diagnostic categories were performed using the Kruskal–Wallis test. Pairwise comparisons between groups were conducted using the Mann–Whitney U test when appropriate. Spatial comparisons between tumor center and invasive front immune densities were evaluated using the Wilcoxon signed-rank test.

Correlations between immune parameters and clinicopathological variables were assessed using Spearman correlation analysis. A P-value < 0.05 was considered statistically significant.

## Results

A total of 90 gallbladder specimens were included in the study cohort. Cases were collected from multiple collaborating hospitals and centrally analyzed at the Department of Molecular Pathology, TSMU. The cohort encompassed inflammatory lesions, precursor epithelial alterations, and invasive carcinoma, allowing evaluation of immune microenvironment changes across the full spectrum of gallbladder pathology. The distribution of cases across diagnostic categories was designed to represent inflammatory, premalignant, and malignant stages of gallbladder carcinogenesis.

The diagnostic distribution included 40 cases of invasive gallbladder adenocarcinoma, 10 cases of chronic cholecystitis, 10 cases of intestinal metaplasia, and 30 cases of biliary intraepithelial neoplasia representing equal numbers of BilIN-1, BilIN-2, and BilIN-3 lesions. Thus, inflammatory and precursor lesions together accounted for 50 cases, whereas invasive carcinoma accounted for 40 cases.

The mean patient age at diagnosis was 60.7 years (standard deviation 8.9; range 45–70 years). The cohort consisted of 50 female patients and 40 male patients. This distribution reflects the recognized predominance of gallbladder pathology among women in many populations (Table 3).

**Table 3:** Clinicopathological Characteristics of the Study Cohort

Parameter	Chronic cholecystitis (n = 10)	Intestinal metaplasia (n = 10)	BilIN (n = 30)	Invasive carcinoma (n = 40)	Total cohort (n = 90)
Age, mean ± SD (years)	59.3 ± 8.1	58.7 ± 7.5	60.9 ± 9.2	62.1 ± 8.8	60.7 ± 8.9
Female sex, n (%)	7 (70%)	6 (60%)	17 (57%)	20 (50%)	50 (55.6%)
Male sex, n (%)	3 (30%)	4 (40%)	13 (43%)	20 (50%)	40 (44.4%)
Cholelithiasis present, n (%)	8 (80%)	7 (70%)	18 (60%)	24 (60%)	57 (63%)
Specimen type: cholecystectomy, n (%)	10 (100%)	10 (100%)	30 (100%)	40 (100%)	90 (100%)
High-grade dysplasia (BilIN-3), n (%)	–	–	10 (33%)	–	–
Tumor grade (G2–G3), n (%)	–	–	–	28 (70%)	–
Lymphovascular invasion, n (%)	–	–	–	14 (35%)	–
Perineural invasion, n (%)	–	–	–	10 (25%)	–
Liver metastasis during follow-up, n (%)	–	–	–	8 (20%)	–

Clinical follow-up data included recurrence, metastasis, and survival status. Liver metastases were recorded in a subset of carcinoma cases during the follow-up period and were used for comparative immune microenvironment analysis.

Histological evaluation confirmed a morphological continuum consistent with the established sequence of gallbladder carcinogenesis. Chronic cholecystitis specimens demonstrated mucosal hyperplasia, dense lymphoplasmacytic inflammatory infiltrates, and varying degrees of epithelial reactive atypia.

Intestinal metaplasia cases showed replacement of the native biliary epithelium by intestinal-type epithelium containing goblet cells and absorptive epithelial cells. These lesions were frequently associated with chronic inflammatory changes within the lamina propria.

**Figure 2:**
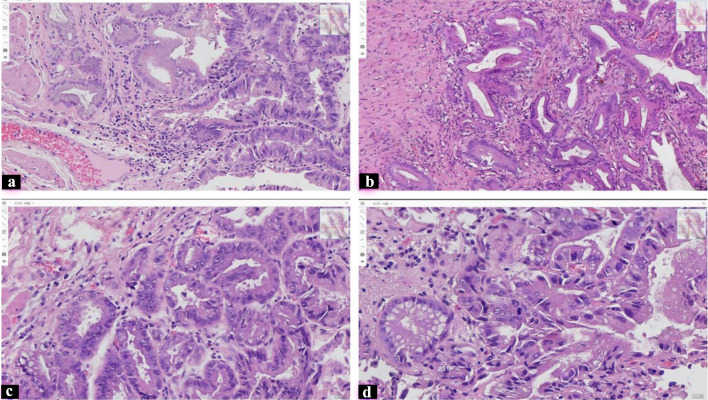
Histological spectrum of gallbladder epithelial lesions. Representative hematoxylin–eosin (H&E) stained sections illustrating the morphological progression from inflammatory to dysplastic gallbladder lesions. (a) Chronic cholecystitis showing inflammatory mucosa with prominent lymphoid infiltration. (b) Biliary intraepithelial neoplasia grade 1 (BilIN-1) characterized by mild epithelial atypia and preserved glandular architecture. (c) Biliary intraepithelial neoplasia grade 2 (BilIN-2) demonstrating moderate nuclear atypia and epithelial stratification. (d) Biliary intraepithelial neoplasia grade 3 (BilIN-3/carcinoma in situ) with marked nuclear pleomorphism, loss of polarity, and pronounced epithelial dysplasia. All images were obtained at × 400 magnification.

Biliary intraepithelial neoplasia lesions demonstrated progressive epithelial dysplasia characterized by increasing nuclear atypia and architectural distortion. BilIN-1 lesions exhibited mild nuclear enlargement and minimal stratification, whereas BilIN-2 lesions showed moderate cytological atypia with increased nuclear stratification. BilIN-3 lesions demonstrated marked nuclear pleomorphism and architectural disorganization consistent with carcinoma in situ (Fig. 2).

**Figure 3:**
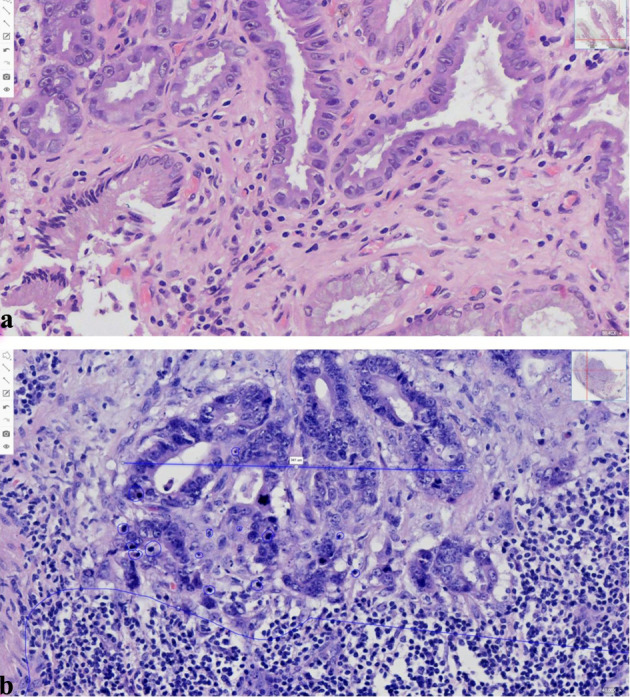
Inflammatory microenvironment in non-neoplastic and malignant gallbladder lesions. Representative whole-slide images (WSI) of hematoxylin–eosin (H&E) stained gallbladder tissue at × 500 magnification. (a) Chronic cholecystitis demonstrating foci of Inflammatory mucosa with minimal lymphocytic infiltration within the lamina propria and reactive epithelial changes. (b) Gallbladder adenocarcinoma showing invasive tumor glands surrounded by a dense peritumoral inflammatory infiltrate. The tumor region is highlighted, and intratumoral lymphocytes are indicated to illustrate the immune cell distribution within the tumor microenvironment.

Invasive gallbladder adenocarcinoma was characterized by irregular glandular or papillary structures infiltrating the gallbladder wall. Tumor cells demonstrated variable nuclear pleomorphism and mitotic activity. A desmoplastic stromal reaction was frequently observed in association with invasive tumor glands (Fig. 3).

### Immune microenvironment across the gallbladder lesion spectrum

Quantitative immunohistochemical analysis revealed substantial variability in immune cell infiltration across gallbladder lesions.

Across the entire cohort, CD8-positive cytotoxic T lymphocytes demonstrated a mean density of approximately 331 cells/mm^2^. The distribution of CD8-positive lymphocytes varied widely among cases, ranging from 30 to 1,000 cells/mm^2^ (Table 4). In inflammatory lesions such as chronic cholecystitis, CD8-positive lymphocytes were commonly observed within the lamina propria and surrounding epithelial structures. In dysplastic and malignant lesions, cytotoxic lymphocytes were frequently present within the tumor stroma but showed heterogeneous distribution. Values are presented as median (IQR). Immune cell ratios were calculated using median densities for each diagnostic category.

**Table 4:** Quantitative Immune Microenvironment Parameters Across Gallbladder Lesion Spectrum

Marker (cells/mm^2^)	Chronic cholecystitis	Intestinal metaplasia	BilIN	Carcinoma	P-value
CD8 cytotoxic lymphocytes	350 (120–620)	320 (110–590)	300 (90–540)	290 (80–520)	0.041
FOXP3 regulatory T cells	180 (60–320)	210 (70–350)	260 (110–430)	320 (140–520)	< 0.001
CD163 macrophages	520 (240–800)	640 (320–920)	760 (400–1,200)	880 (450–1,800)	< 0.001
CD8/FOXP3 ratio	1.94	1.52	1.15	0.91	0.006
CD8/CD163 ratio	0.67	0.50	0.39	0.33	0.012

FOXP3-positive regulatory T cells demonstrated a mean density of approximately 261 cells/mm^2^ across the cohort. In inflammatory lesions, FOXP3-positive cells were generally less abundant. In contrast, dysplastic lesions and invasive carcinoma demonstrated increased densities of regulatory T cells, suggesting progressive enrichment of immunosuppressive immune populations during gallbladder carcinogenesis.

CD163-positive macrophages represented the most abundant immune cell population within the immune landscape. The mean macrophage density across all cases was approximately 733 cells/mm^2^, with values reaching up to 1,800 cells/mm^2^ in highly infiltrated tumors. Macrophages were frequently concentrated within the tumor stroma and surrounding invasive tumor glands.

Overall, these findings indicate progressive remodeling of the immune microenvironment characterized by increased representation of immunosuppressive immune populations in advanced lesions.

### Spatial immune architecture in gallbladder adenocarcinoma

Spatial immune analysis was performed in carcinoma cases by digitally defining tumor center and invasive tumor front regions on whole-slide images.

**Figure 4:**
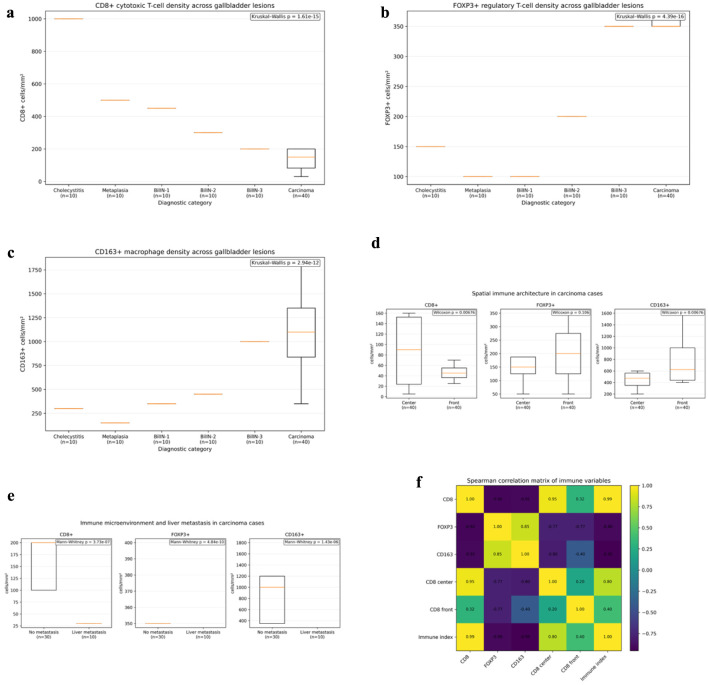
Quantitative and spatial characterization of immune cell populations across gallbladder inflammatory, premalignant, and malignant lesions. (a) CD8^+^ cytotoxic T-cell density across chronic cholecystitis, intestinal metaplasia, biliary intraepithelial neoplasia (BilIN-1–3), and invasive carcinoma. (b) FOXP3^+^ regulatory T-cell density across the same lesion spectrum. (c) CD163^+^ macrophage density across diagnostic categories. (d) Spatial distribution of immune cells in carcinoma, comparing tumor center and invasive front. (e) Comparison of immune cell densities between non-metastatic and liver-metastatic carcinomas. (f) Spearman correlation matrix showing relationships among immune variables and the composite immune index.

Cytotoxic CD8-positive lymphocytes demonstrated a characteristic spatial distribution pattern. In many tumors, cytotoxic CD8-positive lymphocytes accumulated preferentially at the invasive tumor margin, whereas the tumor center demonstrated comparatively lower CD8-positive lymphocyte density. In contrast, the tumor center frequently demonstrated reduced cytotoxic lymphocyte infiltration (Fig. 4).

FOXP3-positive regulatory T cells were detected in both tumor compartments but often showed enrichment in stromal regions adjacent to invasive tumor glands (Table 5). Similarly, CD163-positive macrophages were particularly abundant at the tumor–stroma interface and within desmoplastic stromal areas surrounding invasive carcinoma.

**Table 5:** Spatial Immune Architecture in Invasive Gallbladder Carcinoma

Marker	Tumor center (cells/mm^2^)	Invasive tumor front (cells/mm^2^)	P-value
CD8 cytotoxic T lymphocytes	220 (80–420)	410 (160–760)	< 0.001
FOXP3 regulatory T cells	290 (120–480)	340 (150–540)	0.031
CD163 macrophages	780 (420–1,320)	920 (510–1,500)	0.018

This spatial organization suggests the presence of a complex immune regulatory microenvironment at the invasive tumor margin, where both cytotoxic and immunosuppressive immune populations coexist.

Composite immune indices were calculated to integrate information from multiple immune cell populations and provide an overall representation of immune microenvironment balance.

The Immune Index demonstrated increasing values across the progression from inflammatory lesions to invasive carcinoma. Higher index values were generally observed in malignant tumors compared with non-neoplastic lesions, indicating relative dominance of immunosuppressive immune populations within the tumor microenvironment.

These composite indices provide a simplified quantitative measure of immune balance within gallbladder lesions and may facilitate comparison between cases with differing immune profiles.

### Immune microenvironment and metastatic behavior

Comparative analysis was performed between carcinoma cases with and without liver metastasis during follow-up.

**Figure 5:**
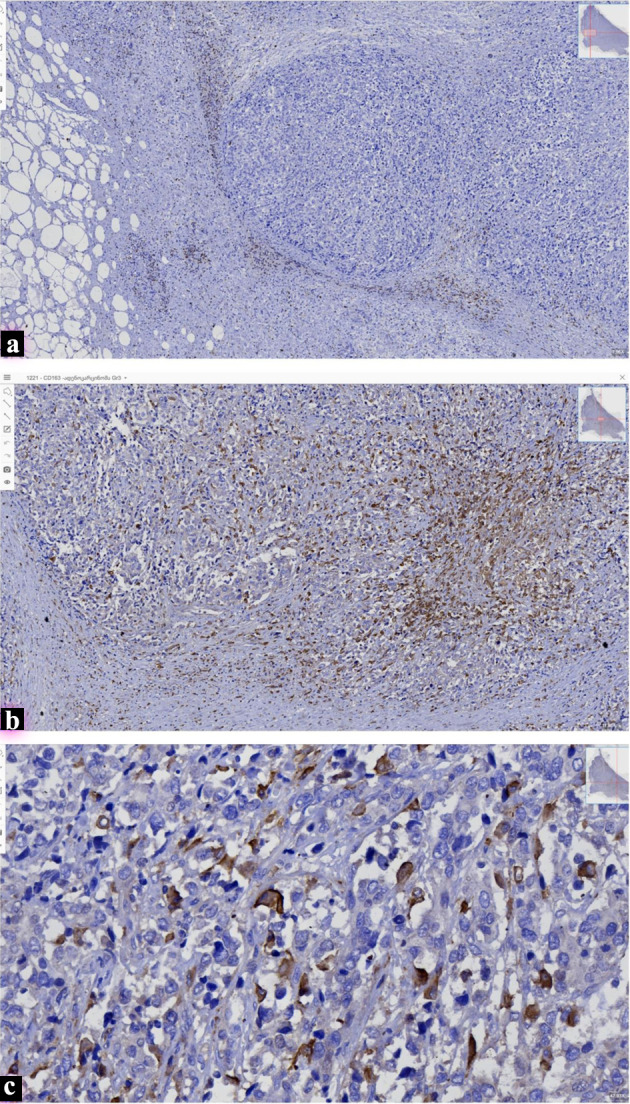
Immune cell infiltration in gallbladder adenocarcinoma and metastatic lesions. Representative immunohistochemical staining demonstrating immune cell populations associated with gallbladder adenocarcinoma. (a) CD8 immunostaining showing cytotoxic T-lymphocyte infiltration within a metastatic liver nodule in a patient with a clinical history of biliary adenocarcinoma (× 40). (b) CD163 immunohistochemistry highlighting tumor-associated macrophages within the metastatic liver lesion (× 300). (c) High-power view demonstrating abundant CD163-positive macrophages within the tumor center of poorly differentiated gallbladder adenocarcinoma (G3) (× 450).

Tumors associated with metastatic spread tended to demonstrate lower densities of CD8-positive cytotoxic lymphocytes and increased infiltration by FOXP3-positive regulatory T cells and CD163-positive macrophages (Fig. 5). This pattern suggests a shift toward an immunosuppressive immune landscape in metastatic tumors (Table 6).

**Table 6:** Immune Microenvironment and Metastatic Behavior in Gallbladder Carcinoma

Marker	Non-metastatic tumors (n = 32)	Metastatic tumors (n = 8)	P-value
CD8 density (cells/mm^2^)	310 (150–580)	210 (80–340)	0.012
FOXP3 density (cells/mm^2^)	300 (120–480)	390 (210–650)	0.009
CD163 density (cells/mm^2^)	820 (420–1,280)	1,040 (600–1,650)	0.015
Immune suppression index	1.35	1.92	0.004

Consequently, metastatic tumors demonstrated higher composite immune index values compared with non-metastatic tumors (Table 6). These findings indicate that enrichment of immunosuppressive immune populations within the tumor microenvironment may be associated with increased metastatic potential in GBC.

## Discussion

The present multicenter study provides a quantitative characterization of the immune microenvironment across the spectrum of gallbladder lesions using digital pathology and IHC. The results demonstrate progressive remodeling of immune cell populations during gallbladder carcinogenesis, with increasing infiltration of immunosuppressive immune components and spatial redistribution of cytotoxic lymphocytes in invasive carcinoma. These findings support the concept that immune microenvironment alterations accompany the transition from inflammatory and precursor epithelial lesions to invasive gallbladder cancer.

Cytotoxic CD8-positive T lymphocytes represent a central component of antitumor immune responses. In the present cohort, CD8-positive lymphocytes were detected in all diagnostic categories but demonstrated heterogeneous density in malignant lesions. This variability is consistent with observations in other gastrointestinal malignancies where the degree of cytotoxic lymphocyte infiltration reflects differences in tumor–immune interaction. Reduced cytotoxic immune dominance within invasive carcinoma may reflect impaired immune surveillance during tumor progression [9, 10].

In contrast to the variability observed for CD8-positive lymphocytes, regulatory T cells and macrophages demonstrated progressive enrichment in advanced lesions. FOXP3-positive regulatory T cells suppress cytotoxic immune activity and contribute to tumor-associated immune tolerance. Their increased presence in dysplastic lesions and invasive carcinoma observed in this study is consistent with findings reported in hepatobiliary and gastrointestinal cancers [11]. Regulatory T-cell expansion within the immune landscape may therefore represent an important mechanism of immune escape during gallbladder tumorigenesis.

Similarly, CD163-positive macrophages represented the most abundant immune cell population within the tumor microenvironment. Macrophages with an M2-like phenotype have been associated with tumor-promoting functions including angiogenesis, stromal remodeling, and suppression of cytotoxic immune responses [12, 13]. The marked increase in macrophage density observed in carcinoma cases supports the concept that macrophage-mediated immune modulation contributes to the tumor-supportive microenvironment of GBC. Similar enrichment of CD163-positive tumor-associated macrophages has been reported in other hepatobiliary malignancies, including intrahepatic cholangiocarcinoma, where macrophage density correlates with tumor aggressiveness and poor prognosis. Recent single-cell transcriptomic studies of GBC have similarly demonstrated development of an immunosuppressive microenvironment characterized by regulatory T cells, exhausted CD8-positive lymphocytes, and tumor-associated macrophages [14, 15]. These observations support the concept that macrophage-mediated immune modulation represents a common feature of hepatobiliary immune landscape. The present findings extend these observations to gallbladder carcinogenesis.

In addition to immune cell composition, the spatial distribution of immune infiltrates provides important information regarding tumor–immune interactions. Spatial analysis in the present study demonstrated preferential localization of CD8-positive lymphocytes at the invasive tumor margin with comparatively lower infiltration of the tumor center. This pattern is consistent with immune exclusion, a phenomenon described in several solid tumors where cytotoxic lymphocytes accumulate at the tumor–stroma interface but fail to effectively penetrate tumor tissue [8]. Similar immune exclusion patterns have been described in multiple solid tumors including colorectal, pancreatic, and hepatobiliary carcinomas. Immune exclusion may reflect stromal barriers, chemokine gradients, or local immunosuppressive signaling within the tumor microenvironment.

The coexistence of cytotoxic lymphocytes with regulatory T cells and macrophages at the invasive tumor margin suggests the presence of a complex immune regulatory niche. Although cytotoxic immune cells are present within the immune landscape, their antitumor activity may be attenuated by neighboring immunosuppressive populations. This balance between cytotoxic and suppressive immune cells represents a key determinant of tumor immune dynamics.

To integrate multiple immune parameters, composite immune indices were calculated. Increased immune suppression indices in invasive carcinoma and metastatic tumors reflect the relative dominance of immunosuppressive immune populations within the immune landscape. Although these indices require validation in independent cohorts, they provide a simplified quantitative representation of immune balance that may be useful for comparing immune microenvironments across tumor categories.

The association between immune landscape alterations and metastatic behavior represents an important observation of this study. Tumors associated with liver metastases demonstrated reduced cytotoxic lymphocyte infiltration together with increased regulatory T-cell and macrophage densities. Similar immune profiles have been reported in other aggressive malignancies and suggest that immunosuppressive immune landscape may facilitate tumor dissemination [16, 17].

The integration of digital pathology represents a methodological strength of the present study. Traditional manual assessment of immune infiltration is limited by interobserver variability and sampling bias. Whole-slide digital analysis allows objective quantification of immune cell densities and spatial distribution across large tissue areas, improving reproducibility and enabling more precise characterization of tumor immune architecture.

The retrospective design and multicenter origin of the cohort introduce potential variability in tissue fixation and clinical data availability. Additionally, molecular profiling was not performed and therefore potential associations between immune microenvironment features and genetic alterations could not be evaluated. Future studies integrating immune architecture analysis with molecular characterization and prospective clinical data may provide deeper insight into the biological mechanisms underlying gallbladder carcinogenesis.

Overall, the findings of this study contribute to the growing understanding of tumor–immune interactions in GBC and highlight the importance of immune landscape remodeling during disease progression.

### Conclusion

This multicenter digital pathology study demonstrates that gallbladder carcinogenesis is accompanied by significant remodeling of the tumor immune microenvironment. Invasive GBC is characterized by enrichment of FOXP3-positive regulatory T cells and CD163-positive macrophages together with spatial redistribution of CD8-positive cytotoxic lymphocytes toward the invasive tumor margin. These findings suggest the development of an immunosuppressive tumor microenvironment with features of immune exclusion.

These findings indicate that progressive immune remodeling accompanies gallbladder tumor development and may contribute to metastatic behavior. Quantitative spatial analysis of immune architecture using digital pathology represents a reproducible approach for evaluating tumor–immune interactions and may facilitate identification of immune-related biomarkers and potential therapeutic targets in GBC.

## Data Availability

The datasets generated and analyzed during the current study are available from the corresponding author on reasonable request.

## Abbreviations

BilIN: biliary intraepithelial neoplasia
CD: cluster of differentiation
CD8: cytotoxic T lymphocyte marker
CD163: marker of M2-type tumor-associated macrophages
EMT: epithelial–mesenchymal transition
FFPE: formalin-fixed paraffin-embedded
FOXP3: forkhead box P3 (regulatory T-cell marker)
H&E: hematoxylin and eosin
HER2: human epidermal growth factor receptor 2
IHC: immunohistochemistry
IQR: interquartile range
ROI: region of interest
SD: standard deviation
TSMU: Tbilisi State Medical University
WSI: whole-slide imaging
CK: cytokeratin
CK17: cytokeratin 17
CK19: cytokeratin 19
CDX2: caudal-type homeobox transcription factor 2
Ki-67: proliferation marker
p53: tumor suppressor protein p53
